# Matrix metalloproteinase‐1 facilitates MSC migration via cleavage of IGF‐2/IGFBP2 complex

**DOI:** 10.1002/2211-5463.12330

**Published:** 2017-11-23

**Authors:** Shou P. Guan, Alan T.L. Lam, Jennifer P. Newman, Kevin L.M. Chua, Catherine Y.L. Kok, Siao T. Chong, Melvin L.K. Chua, Paula Y.P. Lam

**Affiliations:** ^1^ Laboratory of Cancer Gene Therapy, Cellular and Molecular Research Division Humphrey Oei Institute of Cancer Research National Cancer Center Singapore Singapore; ^2^ Division of Radiation Oncology National Cancer Center Singapore Singapore; ^3^ Cancer and Stem Cells Biology Program Duke‐NUS Graduate Medical School Singapore Singapore; ^4^ Department of Physiology Yong Loo Lin School of Medicine National University of Singapore Singapore; ^5^ Oncology Academic Program Duke‐NUS Graduate Medical School Singapore Singapore; ^6^ Present address: BTI A*STAR Centros Singapore; ^7^ Present address: Lonza Biologics Tuas Pte Ltd Singapore

**Keywords:** IGF‐1R, IGF‐2, IGFBP2, matrix metalloproteinase 1, mesenchymal stem cells, migration

## Abstract

The specific mechanism underlying the tumor tropism of human mesenchymal stem cells (MSCs) for cancer is not well defined. We previously showed that the migration potential of MSCs correlated with the expression and protease activity of matrix metalloproteinase (MMP)‐1. Furthermore, highly tumor‐tropic MSCs expressed higher levels of MMP‐1 and insulin‐like growth factor (IGF)‐2 than poorly migrating MSCs. In this study, we examined the functional roles of IGF‐2 and MMP‐1 in mediating the tumor tropism of MSCs. Exogenous addition of either recombinant IGF‐2 or MMP‐1 could stimulate MSC migration. The correlation between IGF‐2, MMP‐1 expression, and MSC migration suggests that MMP‐1 may play a role in regulating MSC migration via the IGF‐2 signaling cascade. High concentrations of IGF binding proteins (IGFBPs) can inhibit IGF‐stimulated functions by blocking its binding to its receptors and proteolysis of IGFBP is an important mechanism for the regulation of IGF signaling. We thus hypothesized that MMP‐1 acts as an IGFBP2 proteinase, resulting in the cleavage of IGF‐2/IGFBP2 complex and extracellular release of free IGF‐2. Indeed, our results showed that conditioned media from highly migrating MSCs, which expressed high levels of MMP‐1, cleaved the IGF‐2/IGFBP2 complex. Taken together, these results showed that the MMP‐1 secreted by highly tumor‐tropic MSCs cleaved IGF‐2/IGFBP2 complex. Free IGF‐2 released from the complex may facilitate MSC migration toward tumor.

**Abbreviations**
APMA: p‐aminophenylmercuric acetate
Ctrl: control
EGFR: epidermal growth factor receptor
EPC: endothelial progenitor cells
GPCR: G‐protein‐coupled receptors
HCC: hepatocellular carcinoma
IGF: insulin‐like growth factor
IGFBP: IGF binding proteins
IRS: insulin receptor substrate
MAPK: mitogen‐activated protein kinase
MMP: matrix metalloproteinase
MSC: mesenchymal stem cell
PI3K: phosphoinositide‐3‐kinase
PKCδ: protein kinase C‐δ
rIGF‐2: recombinant IGF‐2
RT: room temperature
SDF‐1: stromal cell‐derived factor‐1
SF: serum free
TGF‐β: transforming growth factor‐β
TNF‐β: TNF tumor necrosis factor alpha
TRAIL: tumor necrosis factor‐related apoptosis‐inducing ligand
uPA: urokinase plasminogen activator
UT: untreated

In recent years, human bone marrow‐derived mesenchymal stem cells have emerged as a potential approach for the delivery of therapeutic genes for human cancer therapy due to their intrinsic tumor tropism, ease of isolation and expansion, and the susceptibility to a broad range of viral infection, thus serving as potential carriers of therapeutic genes or oncolytic viruses 1, 2, 3, 4, 5, 6. MSCs modified to express tumor necrosis factor‐related apoptosis‐inducing ligand (TRAIL) were capable of clearing lung metastases in the treated animals as compared to nontreated animals 7, presumably due to the combined action of the tumor‐tropic MSCs and the proapoptotic effects of TRAIL expression. Similarly, in a model of hepatocellular carcinoma (HCC), local secretion of an antiangiogenesis factor (pigment epithelium‐derived factor) via MSCs resulted in lower tumor volume, reduced lung metastases, and improved survival through the inhibition of tumor angiogenesis 8. However, the mechanism underlying MSCs tumor tropism is not clearly defined.

The recruitment of MSCs to tumor is thought to be mediated through paracrine signaling loop between the chemoattractants from the tumor microenvironment and the corresponding receptors on MSCs. Conversely, it is also possible that autocrine signaling activates various G‐protein‐coupled receptors (GPCR) and growth factor receptors for signaling through the mitogen‐activated protein kinase (MAPK) and phosphoinositide‐3‐kinase (PI3K) pathways to initiate cell migration (reviewed in 9). These processes involve proteolytic processing of precursor proteins such as adhesion molecules, growth factors, cytokines, and their receptors in the microenvironment by matrix metalloproteinases (MMPs), thus enabling MSCs to extravasate from the circulation and home toward the target tissues. For example, the GPCR known as PAR‐1 could transactivate the epidermal growth factor receptor (EGFR) to promote migration of human colon carcinoma cells 10, gastric cancer cell 11, renal carcinoma cells 12, and human keratinocytes 13. Such cooperation between receptors is not restricted to GPCRs and EGFR. One member of the GPCR family known as CXCR‐4 has been shown to form a complex with IGF‐1R in breast tumor cells, and this interaction allowed IGF‐1 to activate migration signaling pathways through CXCR‐4, G_iα2_, and G_β_ 14. In human MSCs, IGF‐1 and IGF‐2 were reported to direct cell migration of MSCs, a process modulated by IGFBPs 15. Notably, IGF‐1 increased MSC migratory response via the CXCR‐4 receptor signaling 16, 17. However, the mechanism underlying IGF‐2 effect on MSC migration is unknown. The IGF system consists of two ligands (IGF‐1 and IGF‐2), two receptors (IGF‐1R and IGF‐2R), and seven IGFBPs 18. IGFs are involved in the regulation of cell metabolism, growth, survival, differentiation, and migration, depending on the biological settings 18. A study has shown that IGF‐1 produced by genetically modified MSCs could restore new bone fracture formation and partially rescued the lack of fracture healing found in insulin receptor substrate (IRS)‐1‐knockout mice 19, 20, indicating that these IGF‐expressing MSCs may migrate to the site of injury for a repair function. During placental development, IGF‐2 is critical for trophoblast migration 18. IGF2 actions on trophoblast in human placenta are regulated by the IGF‐2R, which can function as both a signaling and clearance receptor 21. The IGF‐2/IGF‐2R system has been shown to play a prominent role in the homing of endothelial progenitor cells (EPC) to the neovascular zone 22. IGF‐mediated cellular processes are partly controlled by its association with its binding proteins IGFBPs, which regulate the bioavailability of free IGFs for interaction with its receptor for downstream signal activation 23, 24, 25. Of note, IGFBP2 is the second most abundant IGFBP 26, 27. The interaction between IGFs and IGFBPs occurs in a stoichiometric fashion; high concentration of IGFBPs can inhibit IGF‐stimulated functions by blocking its binding to its receptors.

Matrix metalloproteinases are one of the proteases that maintain the equilibrium of free and bound IGFs in the biological system 25. Previously, we have compared the gene expression profiles of representative MSC isolates with differential tumor‐tropic capabilities, and found that MMP‐1 and IGF‐2 are highly expressed in MSCs exhibiting greatest tumor‐tropic property 28. Our finding that MMP‐1/PAR‐1 signaling is necessary for MSC tumor tropism is in accordance with the study of Ponte AL and colleagues where IGF is one of the most potent chemotactic factors in stimulating bone marrow‐derived MSCs. Furthermore, the constitutively expressed MMP‐1 in MSCs can be induced upon TNF‐α treatment, indicating that MSC migration may be favored by the increased local concentration of collagenases and gelatinases especially around the inflamed sites 29.

In the present study, we examined the functional roles of IGFs and MMP‐1 in mediating MSC tumor tropism. Our data demonstrated that MMP‐1 secreted by highly tumor‐tropic MSCs could act as IGFBP2 proteinase, resulting in cleavage of the IGF‐2/IGFBP2 complex followed by the extracellular release of free IGF‐2. The unraveling of the signaling mechanism for IGF‐2, IGFBP2, and their interaction with MMP‐1 on MSC migration to tumors may potentially be useful for future manipulation of these cells for targeted therapy.

## Materials and methods

### Cell Culture and RNAi Transfection

Isolation and characterization of MSCs were performed as previously described 30. All human MSCs were cultured in Dulbecco's modified Eagle's medium (DMEM)/F12 with 10% FBS (Life Technologies, Grand Island, NY, USA), ascorbic acid (Sigma‐Aldrich Corp., St. Louis, MO, USA), and normocin (Invivogen, San Diego, CA, USA). A total of eight donors’ MSCs were analyzed (MSC‐1, MSC‐13, MSC‐15, and MSC‐19 belong to the highly migrating MSCs) versus (MSC‐9, MSC‐21, MSC‐22, and MSC‐23 belong to the poorly migrating MSCs). The median age was 56 years. The grouping of MSCs into high versus low migratory activities is determined based on their *in vitro* migratory activities toward conditioned medium derived from tumor cells using *in vitro* migration assay. All MSCs expressed similar cell surface markers and exhibited multilineage differentiation potentials as characterized previously 28.

RNAi transfection was performed using Lipofectamine RNAiMAX (Life Technologies). Stealth negative control (medium GC; Life Technologies) was used as a control. In brief, all RNAi were transfected at a final concentration of 20 nm into 1 × 10^5^ cells cultured in a 6‐well dish (BD Biosciences, NJ, USA) according to the manufacturer's protocol.

### Collection of conditioned media (CM)

To collect the CM from human HCC Huh‐7, 1 × 10^6^ of Huh‐7 cells were seeded in serum‐free (SF) medium in a T75 flask and incubated at 37 °C with 5% CO_2_. An empty T75 flask containing the serum‐free DMEM was treated in a similar manner. The medium was harvested after 48 h, filtered through a 0.22‐μm syringe filter, and centrifuged at 1500 ***g*** at 4 °C to remove cellular debris.

### *In vitro* migration assay

For determining the *in vitro* migratory potential of MSCs, MSCs (1 × 10^4^) were seeded in 24‐well tissue culture insert with an 8‐μm pore size membrane (BD Biosciences, NJ, USA). SF‐CM from Huh7 was added to the bottom wells of migration chamber using SF DMEM as a control. Following 8‐h incubation, migration of MSCs toward SF‐CM from Huh7 was determined by counting the number of propidium iodide (PI)‐stained nuclei on the underside of the membrane under ×200 magnification.

To assess the effect of exogenous recombinant IGF‐2 (rIGF‐2) and anti‐IGF‐2 blocking antibody on MSC migration, Huh‐7 cells were preincubated in SF media containing different concentrations of rIGF‐2 (25, 50, and 100 ng·mL^−1^; R&D Systems, Minneapolis, MN) or anti‐IGF‐2 antibody (2.5 μg·mL^−1^; R&D Systems). After 24 h, the SF‐CM were harvested and added to the bottom wells of the migration chamber. 1 × 10^4^ MSCs were seeded on the upper chamber, and migration was assessed after 8 h.

To evaluate the effect of IGF‐2 knockdown on MSC migration, Huh7 cells were first transfected with control (Ctrl)‐RNAi or IGF‐2‐RNAi. SF‐CM was subsequently harvested and added to the bottom well of a migration chamber. MSC‐13 (1 × 10^4^ cells) was added to the upper chamber. To assess whether the addition of IGF‐2 was able to rescue the knockdown of IGF‐2, rIGF‐2 (200 ng·mL^−1^; R&D Systems) was added to the SF‐CM‐Huh7.

For assessing the effect of IGF‐1R or IGF‐2R knockdown on MSC migration, SF‐CM from Huh7 were added to bottom well of migration chamber. MSC‐13 transfected with Ctrl‐RNAi, IGF‐1R‐RNAi, or IGF‐2R‐RNAi (1 × 10^4^ cells) was added to the upper chamber. Migration of cells was determined at 8 h.

For determining whether the addition of exogenous rIGF‐2 or MMP‐1 was able to rescue the defect in IGF‐1R and IGF‐2R, recombinant MMP‐1 (0.5 μg·mL^−1^; R&D Systems) or IGF‐2 (200 ng·mL^−1^; R&D Systems) was added to the upper chamber.

### Immunoblotting

Equal amounts of protein lysates from MSCs were resolved by either 6% or 15% SDS/PAGE and electroblotted onto polyvinylidene difluoride membrane (Millipore, Darmstadt, Germany). Nonspecific binding sites were blocked for 1 h with PBS containing 5% BSA and 0.1% Tween‐20 at room temperature (RT). The membrane was then immunoblotted against anti‐IGFBP2 clone C‐18 (dilution 1 : 1000; Santa Cruz Biotechnology Inc.), IGF‐2 (dilution 1 : 500; R&D Systems), IGF‐1R and IGF‐2R (dilution 1 : 200; R&D Systems) at 4 °C. Following washing and incubation with either rabbit anti‐goat or goat anti‐mouse horseradish peroxidase‐conjugated secondary antibodies (dilution of either 1 : 5000 or 1 : 10 000; DakoCytomation, Denmark), proteins of interest were visualized with an enhanced chemiluminescence using Western Lightning chemiluminescent kit (Perkin‐Elmer, MA, USA). Normalization was performed using antibody against actin (dilution 1 : 20 000; Thermo Fisher Scientific, Fremont, CA) or tubulin (dilution 1 : 5000; Santa Cruz Biotechnology Inc.). Semiquantitation analysis, to normalize the protein bands against respective loading controls, was performed using Metavue software v6.1 (Molecular Devices, LLC, CA).

### Enzyme cleavage assay

Recombinant human IGFBP2 (400 ng; R&D Systems) was cleaved by activated MMP‐1 (ProSci incorporated, Poway, USA) (substrate/enzyme (S:E) molar ratio ranging from 20 : 1 to 1 : 1) in cleavage buffer containing 150 mm NaCl, 10 mm HEPES (pH7.4), and 5 mm CaCl_2_ at 37 °C for 1 h. Reactions were terminated by the addition of 2 ×  Laemmli sample buffer containing 2‐mercaptoethanol. The reaction solution was boiled and resolved by 15% SDS/PAGE under reducing conditions. EDTA (50 and 100 mm; Life Technologies) was used as an MMP inhibitor and was preincubated with activated MMP‐1 (ProSci incorporated, Poway, USA) in an assay buffer (100 mm HEPES, 44 mm sodium phosphate, 0.1% Triton X‐100, 0.1% BSA, pH 7.4) at 37 °C for 1 h before the addition of rIGFBP2. EDTA‐free protease inhibitor mixture (4% v/v, 1 tablet/mL in H_2_O; Roche Diagnostics, Mannheim, Germany) was used as a negative control. Purified pro‐MMP‐2 and pro‐MMP‐9 were activated as described previously 31 and used at 4 : 1 S:E molar ratio for the final reaction and incubated at 37 °C for 20 h after the addition of rIGFBP2.

To assess the effect of MMP‐1 on IGF‐2/IGFBP2 proteolysis, rIGFBP2 (100 ng; R&D Systems) and IGF‐2 were preincubated at molar ratio of 1 : 5 in an assay buffer (100 mm HEPES, 44 mm sodium phosphate, 0.1% Triton X‐100, 0.1% BSA, pH 7.4) at 37 °C for 1 h. Reactions were terminated and immunoblot analysis was carried out as described before.

### Detection of IGFBP2 proteolysis in MSCs SF‐CM

Equal amount of MSC SF‐CM (20 μg) was incubated with 1 mm p‐aminophenylmercuric acetate (APMA) (Sigma‐Aldrich Corp) or vehicle control (PBS) at 37 °C for 20 h and compared to that of the untreated (UT). Reactions were terminated and 40 μL of each sample was subjected to immunoblotting to detect changes in IGFBP2 levels.

### Statistical analysis

Data are presented throughout this study as means ± standard error of the mean (S.E.M). Statistical significance was evaluated by Student's t‐test, and *P *<* *0.05 was considered significant.

## Results

### IGF signaling is important for MSC migration

**Figure 1:**
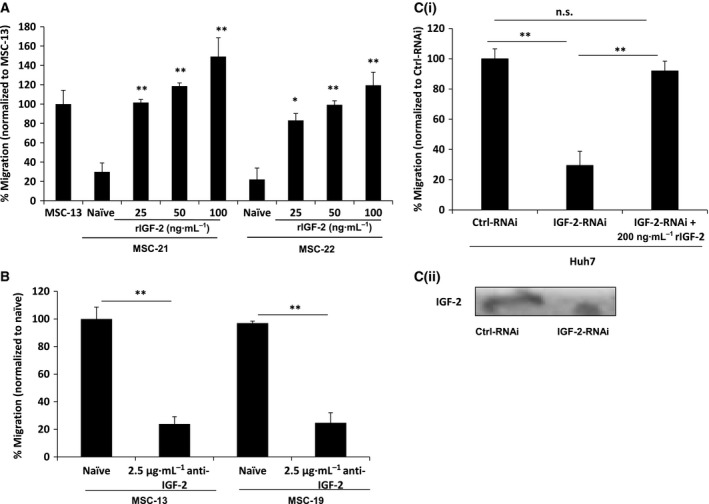
Effects of IGF‐2 on MSC migration. (A) IGF‐2 dose‐dependent stimulation on migration of two representative poorly migrating MSCs (MSC‐21 and MSC‐22). Huh7 was exposed to the specified concentration (25, 50, and 100 ng·mL^−1^) of rIGF‐2, and the migration of MSCs toward this SF‐CM was analyzed using the modified Boyden chamber assay. Bar graph represents percentage of migrated cells normalized to MSC‐13. (B) Effect of anti‐IGF‐2‐blocking antibody on the migration of two highly migrating MSCs (MSC‐13 and MSC‐19) was examined. SF‐CM harvested from Huh7 incubated in the presence of anti‐IGF‐2 was harvested and added to the bottom wells of the migration chamber. IgG was used as negative control. (C) Targeted knockdown of IGF‐2 mRNA inhibited migration of MSCs. Migration of MSC‐13 toward SF‐CM harvested from IGF‐2‐RNAi‐transfected Huh7 cells was analyzed with or without rIGF‐2 using a modified Boyden chamber assay. Bar graph represents percentage of migrated cells normalized to Ctrl‐RNAi. The levels of IGF‐2 proteins were effectively reduced in the respective samples in the presence of respective RNAi treatment, as shown by western blot analysis. For all experiments, percentage of migration was assessed after 8 h. *n.s*, not significant; *, *P* < 0.05; **, *P* < 0.01

Previously, we have demonstrated that not all human bone marrow‐derived MSCs exhibited similar tumor tropism despite identical method of isolation and indistinguishable cell surface phenotypes or multipotent characteristics 28. Apart from MMP‐1, IGF‐2 is the most differentially expressed gene identified when the gene expression profiles of highly migrating MSCs are compared with poorly migrating MSCs by cDNA microarray 28. To determine whether IGF‐2 signaling is important for MSC migration, the migratory potential of poorly migrating MSC‐21 and MSC‐22 toward Huh7‐CM treated with rIGF‐2 was assessed, with MSC‐13 as a positive control 32. Recombinant IGF‐2 (rIGF‐2) stimulated migration of MSC‐21 and MSC‐22 toward Huh7‐CM in dose‐dependent manner (Fig. 1A), corroborated by the reversal of this phenomenon with neutralizing anti‐IGF‐2 antibody. Addition of 2.5 μg·mL^−1^ of neutralizing IGF‐2 antibody to the Huh7‐CM inhibited the migration of the highly migratory MSC‐13 and MSC‐19 by more than 70% (Fig 1B). Next, we examined the possible involvement of IGF‐2 secreted by tumor cells in recruiting MSCs. CM was first harvested from Ctrl‐RNAi‐ and IGF‐2‐RNAi‐transfected Huh7. Targeted knockdown of IGF‐2, as validated by the decrease in IGF‐2 levels in the Huh7‐CM (Fig. 1Cii), resulted in a ~ 70% reduction in the percentage of MSCs migrated toward Huh7‐IGF‐2‐RNAi‐CM (Fig. 1Ci). Impaired MSC migration could be restored through the addition of rIGF‐2 into Huh7‐IGF‐2‐RNAi‐CM. Taken together, these results showed that IGF‐2 signaling is important for MSC migration toward tumor cells.

### IGF/IGFR axis, facilitated by MMP‐1, plays a critical role in MSCs tumor tropism

**Figure 2:**
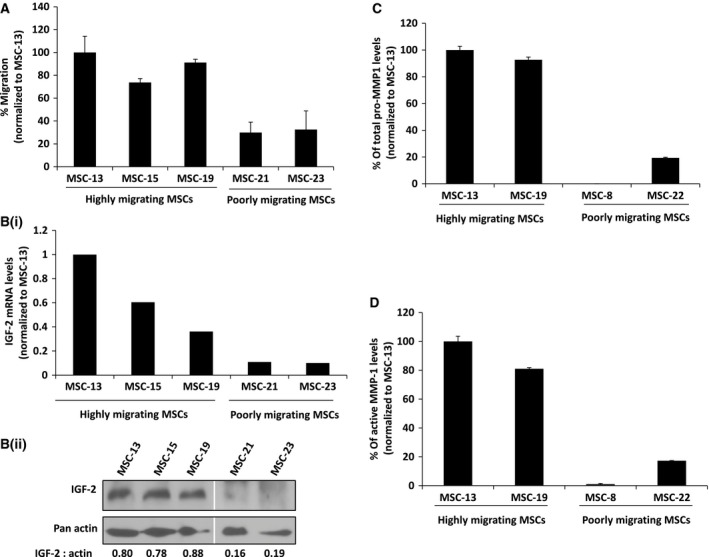
MSC migration correlates with IGF‐2 and MMP‐1 expression. (A) Differential migration abilities of highly migrating MSCs (MSC‐13, MSC‐15, and MSC‐19) and poorly migrating MSCs (MSC‐21 and MSC‐23) toward SF‐CM from Huh7 cells were analyzed using a modified Boyden chamber assay. Bar graph represents percentage of migrated cells using MSC‐13 as control. (Bi) Quantitative real‐time PCR analysis of IGF‐2 mRNA expression levels in highly migrating and poorly migrating MSCs normalized to MSC‐13. (Bii) Western blot analysis on IGF‐2 protein expression levels in highly migrating and poorly migrating MSCs. IGF‐2 bands were semiquantitated against the respective actin controls. (C) Percentage of pro‐MMP‐1 expression levels in 100 μg of CM harvested from various MSCs normalized to MSC‐13. Data shown are the average of triplicate from representative experiments. (D) Percentage of MMP‐1 activity was quantitated from the same amount of CM harvested from various MSCs and normalized to MSC‐13. Data shown are averages of triplicate from representative experiments.

Next, we correlated the migration potential of five selected MSCs isolates with their corresponding IGF‐2 expression levels. As shown in Fig. 2A, MSC‐13, MSC‐15, and MSC‐19 exhibited higher migrating potential toward Huh7‐CM in comparison with MSC‐21 and MSC‐23, which corresponded to increased IGF‐2 mRNA and protein expression in the more migratory primary MSCs (Fig. 2Bi and ii, respectively). The expression and activity of MMP‐1 were determined in two representative MSCs with high and low tumor‐tropic properties. Because we do not have sufficient MSC‐21 that is kept within the passage 4, we have substituted MSC‐21 with another poorly migrating MSC denoted as MSC‐8. The results showed that total pro‐MMP‐1 protein expression (Fig. 2C) and its corresponding MMP‐1 activity (Fig. 2D) were higher in MSCs with greater tumor‐tropic potential in comparison with the poorly migrating MSCs, suggesting possible crosstalk between MMP‐1 and IGF‐2 signaling.

**Figure 3:**
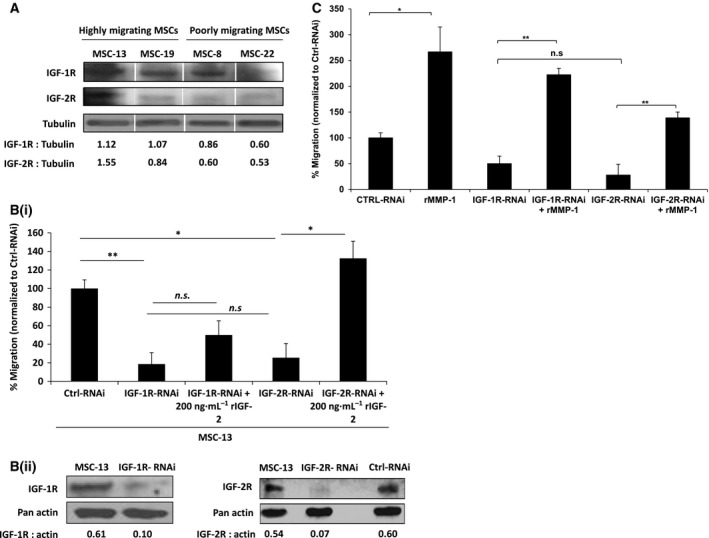
IGF/IGFR axis, facilitated by MMP‐1, plays a critical role in MSC tumor tropism. (A) Western blot analysis was performed on 40 μg of protein extracts from two highly migrating MSCs (MSC‐13 and MSC‐19) and two poorly migrating MSCs (MSC‐8 and MSC‐22) using anti‐IGF‐1R‐ and anti‐IGF‐2R‐specific antibodies. IGF‐1R‐ and IGF‐2R‐specific bands were semiquantitated against respective tubulin controls. (Bi) Migration of IGF‐1R‐ and IGF‐2R‐RNAi‐transfected MSC‐13 was analyzed with or without 200 ng·mL^−1^ of rIGF‐2 using a modified Boyden chamber assay. Bar graph represents percentage of migrated cells normalized to Ctrl‐RNAi. (Bii) Western blot analysis was performed on IGF‐1R‐ and IGF‐2R‐RNAi‐transfected MSC‐13 to confirm the knockdown of respective proteins. Actin was used as a loading control. Semiquantitation was performed as described in (A). (C) Migration of IGF‐1R‐ and IGF‐2R‐RNAi‐transfected MSC‐13 was analyzed with or without 500 ng·mL^−1^ of rMMP‐1 using a modified Boyden chamber assay. Bar graph represents percentage of migrated cells normalized to Ctrl‐RNAi, and the addition of rMMP‐1 served as control. *n.s*, not significant; *, *P* < 0.05; **, *P* < 0.01

IGF‐2 binds to IGF‐1R, IGF‐2R, and the insulin receptor but only during early fetal development 33, 34. To determine whether the migrating potential correlated with IGF receptor expression on MSCs, immunoblot against IGF‐1R and IGF‐2R was performed using whole‐cell lysates harvested from the various MSC isolates. In representative of highly migrating MSCs (MSC‐13 and MSC‐19), expression of both receptors was slightly higher in comparison with poorly migrating MSCs (MSC‐8 and MSC‐22; Fig. 3A). To further investigate the role of IGF receptors in MSC migration, targeted knockdown of IGF‐1R and IGF‐2R was performed on the highly migrating MSC‐13. Subsequent knockdown of both receptors, as validated by the reduced levels of IGF‐1R and IGF‐2R proteins in comparison with Ctrl‐RNAi‐transfected MSC‐13 (Fig. 3Bii), significantly impaired MSC migration toward Huh7‐CM (Fig. 3Bi). The impaired migration in IGF‐1R‐ or IGF‐2R‐silenced MSC‐13 could be rescued by exogenous rIGF‐2 (Fig. 3Bi). Proteolytically active MMPs are often localized to the cell surface in association with its substrates such as growth factor receptors and adhesion receptors 35, 36. Thus, we examined the potential role of IGF‐1R and IGF‐2R in MMP‐1‐mediated MSC migration. The addition of rMMP‐1 significantly increased MSC migration in IGF‐1R‐RNAi‐transfected MSCs but to a lesser extent in IGF‐2R‐RNAi‐treated MSCs (Fig. 3C). Taken together, these findings indicate that both MMP and IGF‐2 are essential for MSC migration.

### MMP‐1‐induced IGFBP2 proteolysis promotes MSC migration

**Figure 4:**
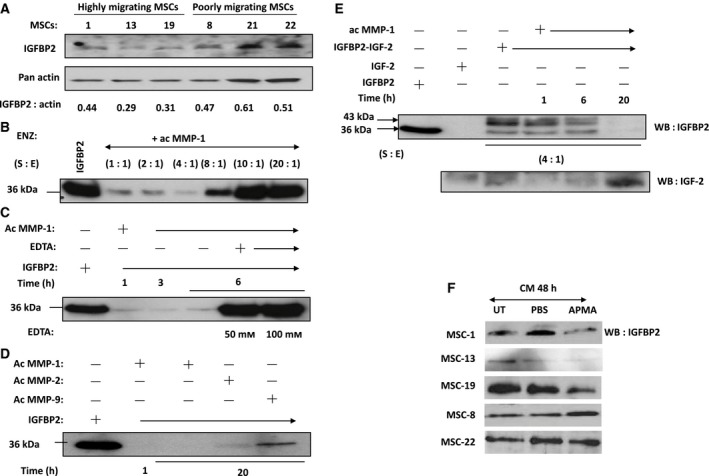
MMP‐1 degrades IGF‐2/IGFBP2 complex to promote MSC migration. (A) Western blot analysis was performed on 40 μg of protein extracts from highly migrating MSCs (MSC‐1, MSC‐13, and MSC‐19) and poorly migrating MSCs (MSC‐8, MSC‐21, and MSC‐22) using anti‐IGFBP2 antibody. Semiquantitation analysis was performed as described previously. (B) Proteolytic activity of active MMP‐1 on human rIGFBP2 (S:E) = (20 : 1) to (1 : 1)] for 1 h. Immunoblotting with anti‐IGFBP2 Ab reveals IGFBP2 with molecular mass of 36 kDa. (C) Time‐course analysis of IGFBP2 degradation. IGFBP2 was incubated with MMP‐1 (S:E) = (4 : 1)] for 1, 3, and 6 h at 37 °C. MMP‐1 was preincubated with EDTA for 1 h prior to the addition of IGFBP2. (D) Proteolytic activities of active MMP‐1, MMP‐2, and MMP‐9 on IGFBP2 for indicated time periods at 37 °C. (E) Time course of proteolytic activity of active MMP‐1 on IGF‐2/IGFBP2 complex (S:E) = (4 : 1)] for 1, 6, and 20 h. Immunoblotting with anti‐IGFBP2 Ab revealed IGFBP2 with molecular mass of 36 kDa or IGF‐2/IGFBP2 complex with molecular mass of 43 kDa and with anti‐IGF‐2 Ab revealed IGF‐2 with mass of 7.5 kDa. (F) To investigate the MMP‐1‐induced IGFBP2 proteolysis, SF‐CM collected from highly migrating MSCs (MSC‐1, MSC‐13, and MSC‐19) and poorly migrating MSCs (MSC‐8 and MSC‐22) was incubated either with PBS or APMA and compared to untreated samples. Effect of APMA‐activated MMPs on IGFBP2 was examined by western blot.

MMP‐9 has been shown to induce IGF‐2/IGFBP2 complex proteolysis resulting in the extracellular release of free IGF‐1 with positive and biological effect on astrocytoma cellular growth and migration 31. To better elucidate the role of MMP‐1/IGF2/IGFBP2 in MSC tumor tropism, we analyzed the expression of IGFBP2 in MSCs with different tumor‐tropic properties by western blot analysis (Fig. 4A). MSCs that do not migrate well to tumor CM (MSC‐8, ‐21, and ‐22) exhibited a slight increase in levels of IGFBP2 in comparison with those with efficient migration potential (MSC‐1, ‐13, and ‐19). Next, we assessed the ability of rMMP‐1 to proteolyze IGFBP2. Using different ratios of IGFBP2 to active MMP‐1, our immunoblot analysis showed that MMP‐1 was able to proteolyze IGFBP2 at the optimal substrate/enzyme ratio of 4 : 1 as shown by the disappearance of the 36‐kDa band (Fig. 4B). By contrast, proteolysis was not observed when the ratio of IGFBP2 to MMP‐1 was increased to 8 : 1, 10 : 1, and 20 : 1. MMP‐1 degrades IGFBP2 in a time‐dependent manner with complete proteolysis observed in 6 h (Fig. 4C). More importantly, the proteolysis activity of MMP‐1 was inhibited when EDTA was added to the reaction, demonstrating that protease activity is required for the degradation of IGFBP2 (Fig. 4C). Next, we examined whether MMP‐2 and mMP‐9 induced migration of MSCs through a paracrine pathway of IGF‐2 release by catalytic cleavage of the IGF‐2/IGFBP2 complex, as opposed to transcriptional regulation 28, 37. Using a previously optimized substrate‐to‐enzyme (S:E) ratio of 4 : 1, MMP‐1 readily proteolyzed IGFBP2 as indicated by the disappearance of the 36‐kDa band in the immunoblot (Fig. 4D). Similarly, addition of activated MMP‐2 and MMP‐9 to the rIGFBP2 protein also resulted in cleavage of the protein, but to a lesser extent. As the molar ratio of S:E for all MMPs was kept constant at 4 : 1 ratio, our results suggested that MMP‐1 was more efficient in IGFBP2 proteolysis than MMP‐9 or MMP‐2 (Fig. 4D).

The ability of MMP‐1 to proteolyze IGF‐2/IGFBP2 complex was further demonstrated by incubating activated MMP‐1 with recombinant IGF‐2/IGFBP2 complex at different time points using S:E molar ratio of 4 : 1. We observed disappearance of the 43‐kDa and 36‐kDa bands, which were indicative of IGF‐2/IGFBP2 complex and free IGFBP2, respectively, after 20 h of treatment (Fig. 4E). Complete proteolysis was evident from the increased expression of the free 7.5‐kDa IGF‐2 band detected on the immunoblot (Fig. 4E). To investigate whether MMP‐1‐induced IGFBP2 proteolysis also occurred in CM derived from MSCs, we treated the MSCs‐CM with APMA followed by immunoblotting for IGFBP2. Activation of MSCs‐CM with APMA reduced the amount of IGFBP2 in the highly migrating MSCs that expressed high level of MMP‐1 (MSC‐1, MSC‐13, and MSC‐19) (Fig. 4F). On the other hand, significant difference was not observed in IGFBP2 expression in the poorly migrating MSC‐8 and MSC‐22. Taken together, these results demonstrated that MMP‐1 secreted by MSCs could efficiently cleave the IGF‐2/IGFBP2 complex, facilitating the migration of MSCs toward tumor cells via the IGF‐2/IGF‐1R signaling axis.

## Discussion

We and others have demonstrated that MMPs and IGFs are involved in MSC tumor tropism 20, 25, 28, 29. In the present study, we further demonstrated that MMP‐1 secreted by the highly tumor‐tropic MSCs cleaved the IGF‐2/IGFBP2 complex that resulted in the release of free IGF‐2, which facilitated MSC migration toward tumor cells via the IGF‐2 signaling axis.

Several lines of evidence suggested that IGF‐2 signaling is required for MSC tumor tropism. The addition of rIGF‐2 enhances the migration of MSCs in a dose‐dependent manner (Fig. 1A), while specific inhibition using antibody against IGF‐2 (Fig. 1B) could attenuate MSC migration. The tumor‐tropic activities of MSC, which could be inhibited by targeted silencing of IGF‐2 in tumor cells, could be fully rescued by the addition of rIGF‐2 (Fig. 1C), suggesting that IGF‐2 is critical for tumor tropism of MSCs, which concurs with the findings of Fiedler and colleagues 10. Further, exogenous rIGF‐2 was able to restore migration in IGF‐2R‐RNAi‐transfected MSCs, but not in IGF‐1R‐RNAi‐transfected cells, demonstrating that IGF‐1R is probably more important for MSC tumor tropism than IGF‐2R. However, we do not exclude the possibility that IGF‐1R may associate with other receptors in mediating the process of MSC migration. For example, the migrating and invasive properties of prostate cancer cells are determined by IGF‐1R activities and receptor tyrosine kinase EGFR under the effects of cadherin molecules 38. IGF‐2R that lacks the tyrosine kinase domain is reported to regulate the bioavailability of IGF‐2 by targeting it to lysosome for degradation 39, thus serving as a regulator of IGF‐2 activities. Interaction between IGF‐2 and IGF‐2R was shown to potentiate mobilization of endothelial progenitor cells through crosstalk with the SDF‐1/CXCR‐4 pathway 22, 40. Other than IGF‐2, IGF‐2R binds to a number of ligands including transforming growth factor‐β (TGF‐β), granzyme B, urokinase plasminogen activator (uPA), glycosylated leukocyte inhibitory factor, and retinoids 41. Interestingly, some of these ligands have been shown to participate in MSC migration 42. For example, TGF‐β, through Smad3 and PKCδ, has been shown to stimulate vascular smooth muscle cell production of monocyte chemoattractant protein (MCP‐1), which is a major chemoattractant for MSCs 43. Histone deacetylase inhibitor has also been shown to increase MSC tumor tropism via induction of uPA expression through ERK activation 44. These findings suggest that the interaction of IGF‐2 or other cellular factors with IGF‐2R may play an important role in MSC migration.

**Figure 5:**
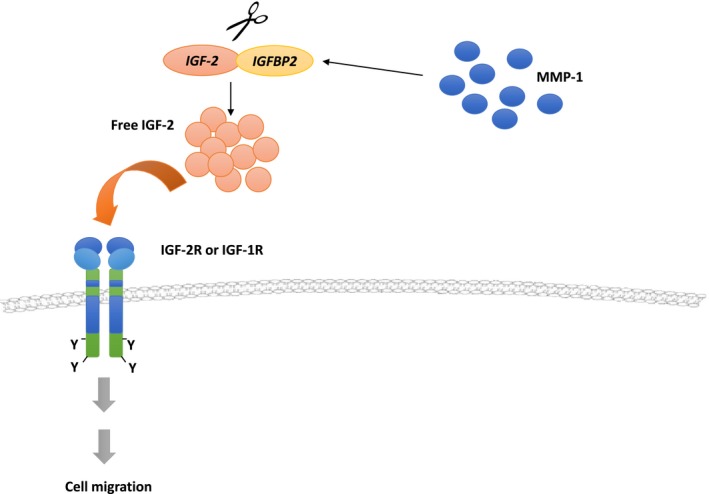
Involvement of MMP‐1 and IGF‐2/IGFBP2 complex in MSC migration. Activated MMP‐1 acts as an IGFBP2 proteinase to induce cleavage of IGF‐2/IGFBP2 complex, thereby increasing the bioavailability of free IGF‐2. Unbound IGF‐2 is then released into the extracellular matrix (ECM) and subsequently initiates the migration of MSCs. Free IGF‐2 can also be released from tumor cells into the ECM, promoting MSC tumor tropism.

Our findings demonstrate that rMMP‐1 could rescue cell migration in IGF‐1R‐RNAi‐transfected MSCs, suggesting that MMP‐1 could exert stimulatory effect, directly or indirectly, on the IGF‐Rs. MMPs are one of the proteases that maintain the equilibrium of free and bound IGFs in the biological system. Circulating IGFs are generally bound to IGFBPs, but can be released through proteolysis of IGFBPs or by binding of IGFBPs to the ECM 25. The potential roles of IGFs and IGFBPs in mediating cell movement and migration have been studied more so in cancer cells than in stromal stem cells, for, for example, degradation of IGF‐1/IGFBP3 complex by MMP‐9‐triggered prostate cancer cell migration 45. Furthermore, Miyamoto *et al*. demonstrated that MMP‐7, which is exclusively expressed in cancer cells, degrades ECM‐bound IGF‐2/IGFBP complex 24. In human bone marrow‐derived MSCs, we have shown that IGF‐2 expression (Fig. 2Bi and Bii) corresponded to MMP‐1 activity (Fig. 3C and 3D) and migration capabilities, hence demonstrating a functional cooperation between two signaling pathways that culminated in cellular motility. As proteolysis of IGFBPs regulates bioavailability of IGFs in tissues, we hypothesized that MMP‐1 could potentially act as IGFBP2 proteinase, resulting in the cleavage of IGF‐2/IGFBP2 complex followed by the extracellular release of free IGF‐2. Indeed, we observed proteolysis of IGFBP2 in a dose‐ and time‐dependent manner by MMP‐1. More significantly, active MMP‐1 readily dissociated the IGF‐2/IGFBP2 complex, resulting in increased levels of free IGF‐2 hence enhancing MSC migration, even in IGF‐1R‐ and IGF‐2R‐knockdown cells (Fig. 5). However, MMP‐1 is not the only MMPs that degrade IGFBP2 as the similar effect was also observed when active MMP‐2 (Fig. 4) and MMP‐9 (Fig. 4; 31) were used. Interestingly, proteolysis of IGFBP2 was not observed by Rorive *et al*. in the setting of astrocytoma 31. MMP‐2 is implicated in stem cell migration and cancer cell invasion 46, 47, 48, 49, 50; thus, perhaps the discrepancy between Rorive *et al*.'s and our results could best be resolved by the idea that IGFBP2 may function independently of IGF 51, 52. IGFBP2 has been shown to promote prostate cancer cell growth through its interaction with integrin, an action that is IGF independent 53. In the case of stem cells, IGFBP2 supported hematopoietic stem cells (HSC) expansion and survival even in IGF signaling‐defective cells, suggesting that IGFBP2 may function in an IGF‐independent manner 54. However, it does not seem to be the case in our system because targeted knockdown of IGF‐2 in the hepatocellular carcinoma cell line Huh7 abolished the migration of MSC‐13, demonstrating that MSC migration toward HCC is dependent on IGF‐2.

In summary, our data suggested a possible mechanism of how MSCs migrate to tumors in that the MMP‐1 produced endogenously by highly migrating MSCs could proteolyze IGF‐2/IGFBP2 complex. Upon cleavage by active MMP‐1, the binding of IGF‐2 to the corresponding receptors could mediate downstream signaling events that lead to cell migration (Fig. 5).

## Author contributions

SPG, ATL, JPN, KLC, MLC, and PYL involved in data analysis and interpretation and manuscript writing. SPG, ATL, JPN, CYK, and STC involved in sample preparation, collection and assembly of data. PYL involved in conception and design, data analysis and interpretation, manuscript writing, financial support, and final approval of manuscript.
